# Supplementary material for: Variation of sugar compounds in Phoebe chekiangensis seeds during natural desiccation
Source: PLoS One. 2024 Mar 7;19(3):e0299669. doi: 10.1371/journal.pone.0299669 (PMC10919866; doi:10.1371/journal.pone.0299669)
Supplement: S1 Table — One-way ANOVA analysis was used for the data. Different lowercase letters represent significant differences at the p < 0.05 level according to the Duncan test. Data represent mean ± SE. “-” means the data is not detected. (DOCX) [file pone.0299669.s001.docx]

**S1 Table. Changes of soluble carbohydrates in seeds of *Phoebe chekiangensis* during desiccation.**

| **Moisture content(%)** | **Starch content**  **(mg·g^-1^ FW)** | **Soluble sugar content**  **(mg·g^-1^ FW)** | **Sucrose**  **content**  **(μg·g^-1^ FW)** | **Trehalose**  **content**  **(μg·g^-1^ FW)** | **Inositol**  **content**  **(μg·g^-1^ FW)** | **Raffinose**  **content**  **(μg·g^-1^ FW)** | **Stachyose**  **content**  **(μg·g^-1^ FW)** | **Fructose**  **content**  **(μg·g^-1^ FW)** | **Glucose**  **content**  **(μg·g^-1^ FW)** | **Galactose**  **content**  **(μg·g^-1^ FW)** | **Arabinose**  **content**  **(μg·g^-1^ FW)** |
| --- | --- | --- | --- | --- | --- | --- | --- | --- | --- | --- | --- |
| 37.06 | 165.88  ± 12.85 bc | 6.73  ± 0.30 e | 24.35  ± 3.10 f | 21.20  ± 2.69 f | 93.24  ± 7.29 abc | - | - | 9883.10  ± 314.60 ab | 10536.09  ± 395.72 bc | 78.76  ± 8.41 cde | 122.49  ± 1.35 bc |
| 33.99 | 167.48  ± 13.79 bc | 7.11  ± 0.21 e | 166.95  ± 10.26 e | 12.96  ± 0.93 g | 87.35  ± 6.67 c | - | - | 9393.37  ± 484.92 bc | 9223.03  ± 371.27 c | 92.98  ± 5.66 bcde | 119.01  ± 1.27 c |
| 30.63 | 346.36  ± 35.63 a | 8.18  ± 0.15 d | 721.64  ± 64.04 c | 33.67  ± 5.30 e | 105.98  ± 4.52 a | - | - | 11703.66  ± 808.91 a | 12401.02  ± 598.57 a | 67.59  ± 5.26 e | 123.19  ± 0.91 bc |
| 28.11 | 190.01  ± 7.64 b | 9.10  ± 0.33 cd | 368.33  ± 46.86 d | 34.48  ± 6.15 e | 87.79  ± 2.06 c | - | - | 10418.38  ± 666.15 ab | 11465.57  ± 695.94 ab | 72.43  ± 7.43 de | 121.56  ± 2.96 c |
| 25.09 | 108.22  ± 3.97 d | 9.94  ± 0.05 bc | 1530.84  ± 84.04 b | 138.96  ± 8.49 c | 98.11  ± 4.73 abc | 158.88  ± 8.60 | - | 11443.25  ± 472.99 a | 12033.18  ± 511.22 ab | 101.40  ± 6.49 abc | 131.61  ± 2.25 a |
| 22.04 | 129.65  ± 3.77 cd | 10.77  ± 0.60 ab | 1691.00  ± 51.19 b | 150.25  ± 9.55 c | 60.93  ± 0.66 d | 165.91  ± 5.40 | - | 8129.40  ± 907.99 c | 11482.75  ± 712.49 ab | 95.87  ± 14.21 bcd | 121.68  ± 3.35 c |
| 19.08 | 129.41  ± 7.60 cd | 10.58  ± 0.14 ab | 1558.67  ± 73.18 b | 67.14  ± 6.47 d | 103.43  ± 1.73 ab | 188.21  ± 11.38 | - | 11490.11  ± 402.33 a | 12611.44  ± 391.82 a | 125.01  ± 9.28 a | 128.69  ± 1.02 ab |
| 16.04 | 119.63  ± 13.73 cd | 10.89  ± 0.37 ab | 9345.17  ± 645.18 a | 213.93  ± 12.94 b | 91.81  ± 3.86 bc | 198.43  ± 9.50 | - | 6068.25  ± 246.79 d | 6965.79  ± 252.72 d | 112.45  ± 5.13 ab | 124.00  ± 1.56 bc |
| 13.05 | 128.39  ± 1.31 cd | 11.85  ± 0.64 a | 10914.51  ± 512.80 a | 509.44  ± 26.52 a | 66.97  ± 0.37 d | 188.41  ± 14.25 | - | 4311.91  ± 290.53 e | 4823.50  ± 351.50 e | 102.14  ± 7.27 abc | 119.98  ± 2.34 c |

One-way ANOVA analysis was used for the data. Different lowercase letters represent significant differences at the *p* < 0.05 level according to the Duncan test. Data represent mean ± SE.

“-” means the data is not detected
