# Supplementary material for: Variation of sugar compounds in Phoebe chekiangensis seeds during natural desiccation
Source: PLoS One. 2024 Mar 7;19(3):e0299669. doi: 10.1371/journal.pone.0299669 (PMC10919866; doi:10.1371/journal.pone.0299669)
Supplement: S1 Fig — The regression line is represented by the black line. GP: Germination percentage, SS: Soluble sugar. (DOCX) [file pone.0299669.s002.docx]

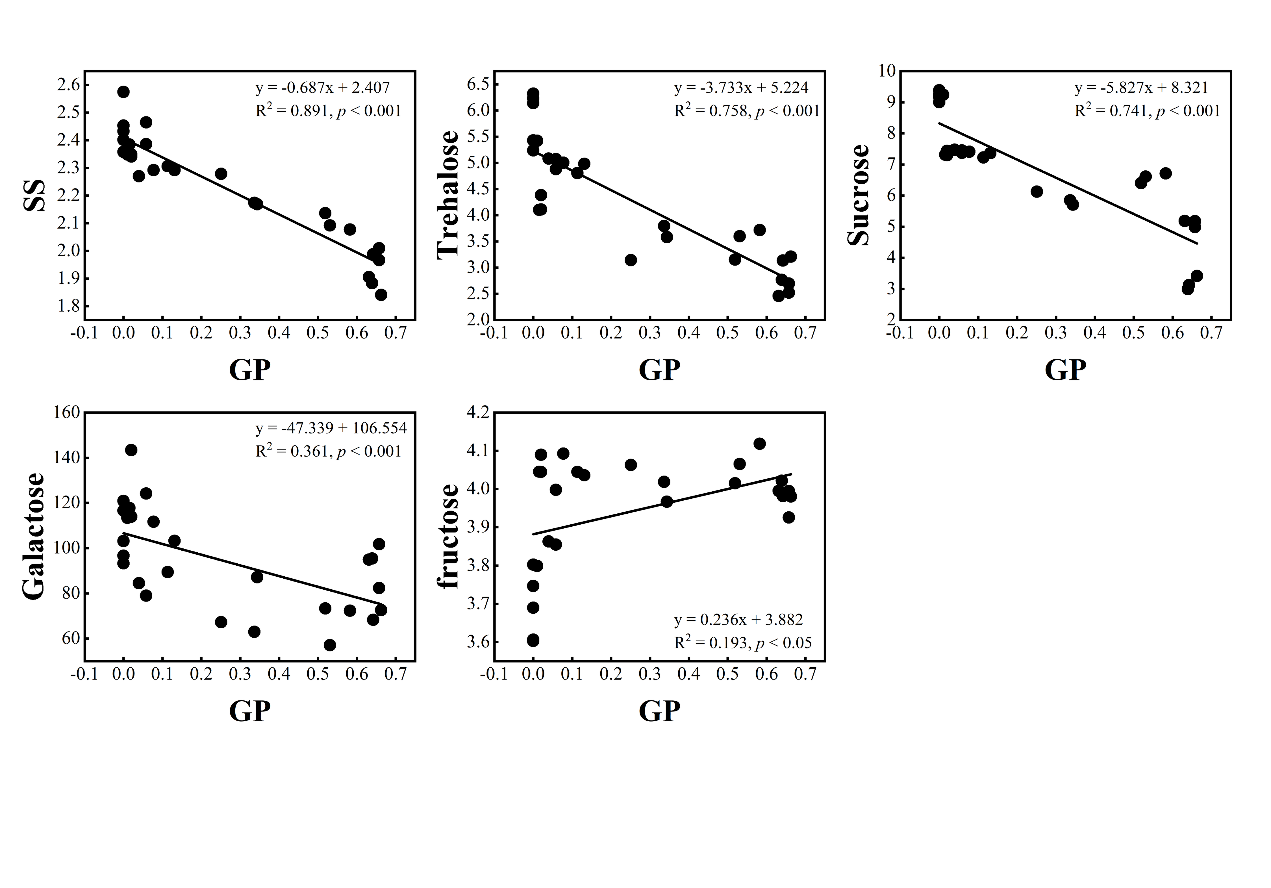


**S1 Fig. Linear regression analysis between germination percentage and some sugar compounds.** The regression line is represented by the black line. GP: germination percentage, SS: soluble sugar.
