# Supplementary material for: Variation of sugar compounds in Phoebe chekiangensis seeds during natural desiccation
Source: PLoS One. 2024 Mar 7;19(3):e0299669. doi: 10.1371/journal.pone.0299669 (PMC10919866; doi:10.1371/journal.pone.0299669)
Supplement: Attachment — Submitted filename: Response to reviewers.docx [file pone.0299669.s003.docx]

**Response to Reviewers**

We gratefully thank the editorial personnel and the reviewers for their efforts on our manuscript. We are glad to have been allowed to revise the manuscript, and we would like to enhance the quality of this manuscript with your constructive comments. Comments are in black and numbered. Our responses are in blue, and quoted texts (including references) from the manuscript are in purple. Please refer to ‘revised manuscript with track changes’ for the ‘line number in the revised manuscript’ mentioned in our reply.

**Academic Editor Comments:** The research is interesting. However, some points are there which must be cleared as mentioned by the reviewers. Discussion part needs refinements. Conclusions should be more precise, meaningful and should be based on data obtained. The overall English grammar of the manuscript needs to be improved. The manuscript can be accepted after these changes and those suggested by the reviewers.

**Reply:** Thank you very much for your comments. As you said, we responded to the questions of reviewers carefully on a one-on-one basis. The discussion section has been improved. The conclusion section has also been revised and is based more on the data obtained. For the English grammar of the manuscript, we did our best to revise it, if there are still grammatical problems, please do not hesitate to contact us, we would very much like to meet your requirements. Once again, thank you for your valuable comments and we hope that the revised manuscript will meet your requirements.

**Additional Editor Comments:** The research is interesting. However, some points are there which must be cleared as mentioned by the reviewers. Discussion part needs refinements. Conclusions should be more precise, meaningful and should be based on data obtained. The overall English grammar of the manuscript needs to be improved. The manuscript can be accepted after these changes and those suggested by the reviewers.

**Reply:** Thank you very much for your comments. As you said, we responded to the questions of reviewers carefully on a one-on-one basis. The discussion section has been improved. The conclusion section has also been revised and is based more on the data obtained. For the English grammar of the manuscript, we did our best to revise it, if there are still grammatical problems, please do not hesitate to contact us, we would very much like to meet your requirements. Once again, thank you for your valuable comments and we hope that the revised manuscript will meet your requirements.

We have also made the corresponding modifications and then uploaded the file according to the requirements of your journal.

**Reviewer 1**

**1. Comment:** The title can be improved. ‘Variation of soluble carbohydrates’ looks a little vague. Perhaps something to reflect the overall results would be good.

**1. Reply:** Thank you very much for your comments on the title of this manuscript. By searching for a relevant reference, we have made the following modifications after consideration:

**Variation of sugar compounds in *Phoebe chekiangensis* seeds during natural desiccation**

**References:**

Zhang Q, Song X, Bartels D. Enzymes and metabolites in carbohydrate metabolism of desiccation tolerant plants. Proteomes. 2016; 4(4):40. <https://doi.org/10.3390/proteomes4040040> PMID: [28248249](https://pubmed.ncbi.nlm.nih.gov/28248249)

**2. Comment:** The writing can be a lot tighter. For example, the first two sentences of the introduction can be combined, and statements like these need reference.

**2. Reply:** Thank you very much for your comments, we have considered and modified the first two sentences of the introduction and cited the references. Also, we have modified sentences 3-4 of the introduction. The modifications are as follows:

*Phoebe chekiangensis* is a second-class protected tree species in China, a newly discovered endemic and endangered species in Tianmu Mountain of China [1], and its economic value is also high [2]. It provides high-quality wood that can be used for high-grade construction, furniture, carvings, and precision molds [2]. It is also widely used in the production of plywood, lacquer, and wooden tires [2].

**References:**

1. Shang C-B. A New Species of the *Phoebe* from Chekiang. Acta Phytotaxon. Sin. 1974; 12(3):295-298.

2.	Li D-L, Jin Y-Q, Shang C-B. Study on cultivation and utilization of rare tree species *Phoebe chekiangensis*. Journal of Jiangsu Forestry Science & Technology. 2004; 31(1):23-25.

**3. Comment:** Can the authors think about giving a range of occurrences for this species?

**3. Reply:** Thank you very much for your comments, we think that giving the range of occurrence of *P. chekiangensis* does help people to understand its ecological characteristics more comprehensively, so we have added the relevant information in the first paragraph of the introduction as follows:

*P. chekiangensis* is distributed in Zhejiang, Fujian, Jiangxi, and southern Anhui Provinces in China, and grows mostly in evergreen broad-leaved forests in hilly valleys or red-soil slopes with an altitude of less than 1000 m, and it prefers a warm, moist climate and acidic or slightly acidic soil [3]. There are broad-leaved evergreen forests with P. chekiangensis as the dominant species in Yunqi and Li’an Temple in Hangzhou, while other areas are scattered [2, 4].

**References:**

2.	Li D-L, Jin Y-Q, Shang C-B. Study on cultivation and utilization of rare tree species *Phoebe chekiangensis*. Journal of Jiangsu Forestry Science & Technology. 2004; 31(1):23-25.

3.	Wu C-P, Zou H-L, Yuan W-G, Huang Y-J, Zhang J, Shen A-H. Investigation and Analysis on the Habitat of *Phoebe chekiangensis*. JOUR. OF ZHEJIANG FOR. SCI. & TECH. 2013; 33(2):1-4.

4.	Xu S-S. Study on population ecology and introduction cultivation of *Phoebe chekiangensis.* Thesis, Nanjing Forestry University. 2004.

**4. Comment:** L43- it is unclear what you mean by breeding technology.

**4. Reply:** Thank you very much for your comments, after our consideration, we believe that the breeding technology is inappropriate in this context, and we have modified this sentence as follows:

***P. chekiangensis* mainly relies on sowing and seedling raising,** and there is a lack of breeding and promotion of excellent strains [6].

**5. Comment:** L48- rising temperatures and more prolonged droughts are weather anomalies; therefore, it is unclear what you mean here.

**5. Reply:** Thank you very much for your comments. Since weather anomalies include more frequent and prolonged droughts and rising temperatures, we have considered and decided to delete the word weather anomalies, and the revised sentence is as follows:

Furthermore, the acceleration of global climate change, such as more frequent and prolonged droughts and rising temperatures, is a serious challenge for recalcitrant species that are highly dependent on specific habitats.

**6. Comment:** L49- didn’t you say this a few lines before?

**6. Reply:** Thank you very much for your comments. We did write a similar repetitive statement in the first few sentences, and after consideration, we decided to delete the previous repetitive statement and then reorganized the sentence L49 with the following modifications:

Therefore, it is crucial to protect the preservation of *P. chekiangensis* germplasm resources.

**7. Comment:** L51- the high moisture content was observed in this study or previous studies. If the former, you should better put them in results.

**7. Reply:** Thank you very much for your comments. We found the fact that *P. chekiangensis* seeds have higher moisture content after abscission and are sensitive to desiccation through pre-experiment. We apologize for not stating it clearly, so we made the following modifications:

**Our pre-experiment found that** *P. chekiangensis* seeds have higher moisture content (MC) after abscission and are sensitive to desiccation.

**8. Comment:** L 52- You have a tendency to repeat the same information using different words. Please combine all these and write concisely.

**8. Reply:** Thank you very much for your comments. Based on your comments, we have made the following modifications:

Our pre-experiment found that *P. chekiangensis* seeds **have higher moisture content (MC) after abscission and are sensitive to desiccation.**

**9. Comment:** L 58- order can be improved. First, talk about desiccation during development, then abscission.

**9. Reply:** Thank you very much for your comments. We also think that the order of improvement here would indeed be better, so we have decided to make the following modifications:

Orthodox seeds undergo maturation desiccation at a later stage of development, and the moisture content of the seeds is low after abscission and can generally be further dried to less than 5% moisture content without damage [11].

**References:**

11. Song S-Q, Liu J, Tang C-F, Cheng H-Y, Wang W-Q, Zhang Q, et al. Research Progress on the Physiology and Its Molecular Mechanism of Seed Desiccation Tolerance. Scientia Agricultura Sinica. 2022; 55(06):1047-1063.

**10. Comment:** L 60- I think storing at -10C is rare; the seeds are usually stored at -18C.

**10. Reply:** Thank you very much for your comments. We referred to the reference listed below [11] and we agree that orthodox seeds are usually stored at -18°C under gene bank conditions, so we decided to modify the storage temperature as follows:

These seeds can be dried to less than 7% MC and stored at **-18°C** for a long time [12-14].

**References:**

Only the new reference added is listed here.

13.	Kijak H, Ratajczak E. What Do We Know About the Genetic Basis of Seed Desiccation Tolerance and Longevity? Int J Mol Sci. 2020; 21(10):3612. <https://doi.org/10.3390/ijms21103612> PMID: [32443842](https://pubmed.ncbi.nlm.nih.gov/32443842)

**11. Comment:** L67- 10% is too low; most recalcitrant seeds die above 10% MC.

**11. Reply:** Thank you very much for your comments. We searched several papers (see below) and found that the moisture content of recalcitrant seeds at the time of death is not easy to define, and people are more inclined to the following two statements: recalcitrant seeds are sensitive to desiccation or recalcitrant seeds are unable to dry to a lower moisture content compared to orthodox seeds. Therefore, after consideration, we have decided to delete this sentence.

~~Unlike orthodox seeds, recalcitrant seeds cannot maintain viability once their moisture content drops below 10% or even 5% [14].~~

**References:**

Kijak H, Ratajczak E. What Do We Know About the Genetic Basis of Seed Desiccation Tolerance and Longevity? Int J Mol Sci. 2020; 21(10):3612. <https://doi.org/10.3390/ijms21103612> PMID: [32443842](https://pubmed.ncbi.nlm.nih.gov/32443842)

Pammenter N, Berjak P. A review of recalcitrant seed physiology in relation to desiccation-tolerance mechanisms. Seed Sci Res. 1999;9(1):13-37. <https://doi.org/10.1017/S0960258599000033>

Perumal A, Varghese B, Govender P, Ramdhani S, Berjak P. Effects of elevated temperatures on germination and subsequent seedling vigour in recalcitrant *Trichilia emetica* seeds. S Afr J Bot. 2014;90:153-162. <https://doi.org/10.1016/j.sajb.2013.11.005>

**12. Comment:** L68- you say previous studies but cited only one reference.

**12. Reply:** Thank you very much for your comments. The citation of the reference here is indeed insufficient, and after consideration, we have decided to add a few references as follows:

Previous studies have indicated that seed acquires desiccation tolerance while accumulating large amounts of soluble sugars during development **[17-20]**.

**References:**

17.	Leprince O, Hendry GAF, McKersie BD. The mechanisms of desiccation tolerance in developing seeds. Seed Sci Res. 2008; 3(4):231-246. <https://doi.org/10.1017/s0960258500001859>

18.	Yang Q-H, Song S-Q, Ye W-H, Yin S-H. Relationship between desiccation-tolerance and sugars in seeds. Bulletin of Botanical Research. 2003; 23(2):204-210.

19.	Koster KL, Leopold AC. Sugars and desiccation tolerance in seeds. Plant Physiol. 1988; 88(3):829-832. <https://doi.org/10.1104/pp.88.3.829> PMID: [16666392](https://pubmed.ncbi.nlm.nih.gov/16666392)

20.	Kuo TM, VanMiddlesworth JF, Wolf WJ. Content of raffinose oligosaccharides and sucrose in various plant seeds. J. Agric. Food Chem. 1988; 36(1):32-36. <https://doi.org/10.1021/jf00079a008>

**13. Comment:** L 69- I think you tend to say important and they are, why not delete ‘are important, and they’

**13. Reply:** Thank you very much for your comments. We think the sentence is more appropriate after your revision, so we decided to delete ‘are important, and they’, and the result of the sentence revision is as follows:

Among the soluble sugars, sucrose and raffinose family oligosaccharides (such as raffinose and stachyose) are considered to be involved in the formation of glass or have a protective effect on membrane phospholipids, or both [21].

**14. Comment:** L73- how?

**14. Reply:** Thank you very much for your question. **We think the sentence (L73) may not be expressed clearly and ‘can cause water in the seeds to transition into a glassy state’ may be better written as ‘can promote the transformation of the cytoplasm into the glassy state during seed desiccation’.** Oligosaccharides can increase the glass transition temperature value (Tg) of the cytoplasm so that the seeds or embryonic axes enter the vitrified state in a higher temperature range [17]. The vitrification of subcellular solution at low moisture content and high sugar water level may limit molecular movement, maintain the static equilibrium of the biochemical activities, and protect the stability of the subcellular in the dry state so that the cytoplasm is protected [17].

**But given the logic of the context, we decided to delete the sentence, and the result is as follows:**

**~~The presence of non-reducing substances such as oligosaccharides can cause water in the seeds to transition into a glassy state.~~**

**References:**

19.	Koster KL, Leopold AC. Sugars and desiccation tolerance in seeds. Plant Physiol. 1988; 88(3):829-832. <https://doi.org/10.1104/pp.88.3.829> PMID: [16666392](https://pubmed.ncbi.nlm.nih.gov/16666392)

**15. Comment:** L75- what do you mean by acquire?

**15. Reply:** Thank you very much for your comments, we think there is a problem with the formulation here. There is a general tendency to state that ‘seed desiccation tolerance is related to the accumulation of sugars [①-④]’. however, for reasons of contextual coherence, we decided to delete this sentence. It reads as follows:

**~~Sucrose, oligosaccharides, and cyclitol are the basic substances for seeds to acquire desiccation tolerance.~~**

**References:**

① Zhu C, Cheng C, Liu X, He J. Accumulation of soluble sugars related to desiccation tolerance during rice (*Oryza sativa* L) seed development and maturation. Seed Sci Technol. 2007; 35(3):649-659. <https://doi.org/10.15258/sst.2007.35.3.12>

② Pammenter N, Berjak P. A review of recalcitrant seed physiology in relation to desiccation-tolerance mechanisms. Seed Sci Res. 1999; 9(1):13-37. <https://doi.org/10.1017/S0960258599000033>

③ Yang X-Q, Jiang X-C, Fu J-R. Soluble Sugar and the Acquisition of Desiccation Tolerance in Peanut (*Arachis hypogaea*) Seeds. Acta Phytophysiologica Sinica. 1998; 24(2):165-170.

④ Ren X-M, Zhu C, Zeng G-W. Current Research of Basic Substances Related to Desiccation Tolerance in Seeds. Chinese Bulletin of Botany. 2001; 18(2):183-189.

**16. Comment:** L77- 106: The paragraph appears too long. I think you could condense the information. Similarly, these are for orthodox seeds. Is there any evidence showing soluble sugar accumulation is different in recalcitrant seeds? If so, you would have to discuss that.

**16. Reply:** Thank you very much for your comments. After some consideration, we have decided to streamline the passage (L77-106) to make it seem less long. In addition, at the end of the paragraph, we added information about soluble sugar changes during the desiccation of recalcitrant seeds (bolded purple font below). Finally, taking the context into account, we placed the information on ‘Changes in hazardous substances such as malondialdehyde and relative conductivity’ (L96-106) in a separate paragraph, later. The result of the modification is as follows:

Pukacka et al. [23] observed that during late development, desiccation-tolerant *Fagus sylvatica* L. seeds exhibited an increase in raffinose content and a decrease in the ratio of sucrose to raffinose, and there was a significant increase in the sucrose/(raffinose + stachyose) ratio and α-galactosidase activity in the seeds after 7 and 12 years of storage. Zhu et al. [24] discovered that subjecting the rice seeds to a slow-drying treatment at different developmental stages could induce sucrose accumulation. Trehalose is one of the most effective sugars for maintaining the structural and functional integrity of membranes and proteins at low water concentrations [25]. Coutinho et al. [26] found that among yeasts that naturally produce trehalose, mutant yeast strains with higher trehalose synthase activity had a greater ability to survive under drying and freezing conditions. Similarly, Bellaloui et al. [27] observed a significant increase in the concentration of stachyose, along with a significant decrease in the concentration of glucose, fructose, and sucrose, in Soybean seeds under water stress and high temperatures. **Kristina and Sharon [28] found that the sucrose content of cotyledons of *Quercus alba* was increased in the later stages of drying. The soluble sugar content was increased in recalcitrant seeds of *Quercus variabilis* [29] during drying.**

**17. Comment:** L107- Perhaps you need to specifically state the objectives of the study here. It looks like you have only investigated the soluble carbohydrates in this study.

**17. Reply:** Thank you very much for your comments. Based on your comments, we have made the following modifications (see purple text below ①). In addition, our team also investigated the physiological responses of *P. chekiangensis* seeds under natural desiccation conditions, including some antioxidant enzymes, soluble protein, free proline, ascorbic acid, reductive glutathione, and endogenous hormones. In this manuscript, we focused on the changes in sugar compounds in seeds during desiccation. Unfortunately, we did not publish all the results in this manuscript and our team hopes that other results will be published later. Thank you again for your valuable comments.

**①:** To the best of our knowledge, current research on *P. chekiangensis* seeds has focused on seed dormancy and measures to break dormancy [7, 8], as well as seed germination characteristics [33], while research on the response of *P. chekiangensis* seeds to desiccation has hardly been reported. Therefore, in this study, we systematically investigated the changes in seed germination, sugar compounds, malondialdehyde, and relative electrical conductivity during the natural drying of the seeds in a room, **focusing on the role of sucrose, oligosaccharides, and hexoses (glucose and fructose) during seed desiccation,** to provide additional data and theoretical support for the sugar metabolism mechanism of desiccation-sensitive seeds.

**References:**

7.	Shi X-H, Shi Z-L. Preliminary study on dormancy physiology of *Phoebe chekiangensis* seeds. Journal of Zhejiang Forestry College. 1990; 7(4):85-90.

8.	Shi X-H, Shi Z-L. Study on Seed and Dormancy of *Phoebe chekiangensis* and Its Releasing Ways. Seed. 1990; (3):76. <https://doi.org/10.16590/j.cnki.1001-4705.1990.03.073>

33.	Li Z, Wang S-J, Liu C-L, Lin X-Z. Study on seed germination characteristics of *Phoebe sheareri* and *Phoebe chekiangensis*. Northern Horticulture. 2012; (7):58-60.

**18. Comment:** L121- the collection time (maturity time) indicates the seeds are naturally dispersed before winter. Last year, when I visited Jiangsu, it was early December, and winter had begun already. It would be good to include more details about dispersal.

**18. Reply:** Thank you very much for your comments, we think this is a good proposal. **The seed maturation period of *P.chekiangensis* is about from late October to mid-November, and the seed dispersal period is about from late November to mid-December [34, 35].** In addition, according to our observation, in Jiangsu, such as Nanjing, the annual winter cooling time is different, early or late, Qionglai City (seed collecting area) is also the case.

**Finally, based on your comments, we have added information on the dispersal of *P. chekiangensis* seeds as follows:**

**The seed maturation period of *P.chekiangensis* is about from late October to mid-November, and the seed dispersal period is about from late November to mid-December [34, 35].**

**References:**

34. State-owned Forest Farm and Forest Seedling Work Station of State Forestry Administration, editor. Seeds of Woody Plants in China. 1st ed. Beijing: Chinese Forestry Publishing House; 2000.

35. Shen G-F, editor. Silviculturetechnic for the Principal Treespecies of China. 2nd ed. Beijing: Chinese Forestry Publishing House; 2020.

**19. Comment:** L126- for how long?

**19. Reply:** Thank you very much for your comments. Normally, we keep the seeds in wet sand for 3-5 months at 4℃. Because in production, after a few months of low-temperature storage, the seeds can be sown in March of the second year [35].

**References:**

35. Shen G-F, editor. Silviculturetechnic for the Principal Treespecies of China. 2nd ed. Beijing: Chinese Forestry Publishing House; 2020.

**20. Comment:** L 126- MC results move to results. However, what do you mean by the low-constant oven drying method? Please specify the temperature and time.

**20. Reply:** Thank you very much for your comments. According to your comments, we moved the moisture content result to the result (L411 in the revised manuscript). **Regarding the determination of seed MC by the low constant temperature oven drying method [36, 37], our explanation is below:**

We sliced 20 intact seeds of *P. chekiangensis*, with each seed evenly cut into 4 slices. We weighed the weight of the dried aluminum box with lid **(W1)**, and then evenly placed 5-6 g of slices into this aluminum box with lid and weighed **(W2)**. After drying the samples in a constant temperature oven **at 103°C for 17h**, we took the samples out and placed them in a desiccator to cool for 30 minutes. After cooling, the weight of the aluminum box with lid and the sample were weighed **(W3)**.

**The seed MC was calculated using the following formula:**

$$MC\left( \% \right)=\frac{W2-W3}{W2-W1}\times100$$

**Finally, after careful consideration, we have revised the sentence (L126) and added a reference to allow for a better understanding of the method of determination of moisture content. The result of the modification is as follows:**

**‘**The initial moisture content of *P. chekiangensis* seeds was measured using the low constant temperature oven drying method **(103 °C for 17 h) [36, 37]**,’

**References:**

36. International Seed Testing Association (ISTA). In: International Seed Testing Association (ISTA), editor. International Rules for Seed Testing. Chapter 9: Determination of Moisture Content. Basserdorf: Switzerland; 2004.

37.	Chen H, Shen Y. Investigation of Water Distribution and Mobility Dynamics in Recalcitrant *Quercus acutissima* Seeds during Desiccation Using Magnetic Resonance Methods. Forests. 2023; 14(4):738. <https://doi.org/10.3390/f14040738>

**21. Comment:** L 128- seed weight results to the results section. What is the 100 seeds method? Please include a reference; if not, describe how this was done.

**21. Reply:** Thank you very much for your comments. Based on your comments, we have moved the seed weight results to the results section (L412 in the revised manuscript). **For the 100 seeds method, we referred to the ‘Forest seed testing procedures (GB 2772-1999)’.** We made the following modifications to the sentence (L128):

‘**and the thousand seed weight was measured using the hundred seeds method [38].’**

**References:**

38. State Bureau of Quality and Technical Supervision. Forest tree seed inspection procedures, GB 2772-1999. Beijing: Standards Press of China, 2000.

**22. Comment:** L133- following formula? I don’t see any formula following, but if you mean the equation given on L149- use the number and refer accordingly.

**22. Reply:** Thank you very much for your comments. As you think, the following formula does refer to the equation at L149. Based on your comments, we have numbered the formula and added a reference to the sentence. The result of the modification is as follows:

According to the following formula (1), the moisture content of the seed lot during desiccation was calculated by the weighing method [39].

Calculation formula of moisture content (MC) [39]:

$R=\frac{M2-M1\times\left( 1-A \right)}{M2}\times100\% (1)$

where: R is the seed moisture content, %; M1 is the mass of the undried seed, g; M2 is the mass of the dried seed, g; A is the initial moisture content of the undried seed, %.

**References:**

39.	Feng J, Shen Y, Shi F, Li C. Changes in seed germination ability, lipid peroxidation and antioxidant enzyme activities of *Ginkgo biloba* seed during desiccation. Forests. 2017; 8(8):286. <https://doi.org/10.3390/f8080286>

**23. Comment:** L 136- I don’t think these were target moisture contents. I would assume you dried the seeds and periodically removed them; if so, why not give time instead of moisture content? I also think ‘portion’ is not a good usage in science. Two seeds can be a portion, and all the seeds except two can also be a portion.

**23. Reply:** Thank you very much for your comments. This is because seed viability is closely related to moisture content in desiccation experiments. When we started designing the desiccation experiment, we tried sampling at 3% moisture content intervals until the moisture content dropped to about 10%, using fresh seeds as a control. However, the moisture contents in the manuscript are actual values during the experiment, which were a little different from the ideally designed moisture contents, but we did not miss important moisture contents in terms of germination percentages. In addition, when we designed the experiment, we were concerned that sampling according to time would tend to miss important moisture contents. Monitoring moisture contents directly according to the weighing method is more feasible for *P. chekiangensis* seeds. Thanks again for your constructive comments.

Based on your comments, we think the word portion is inappropriate and we think it would be better to state the number of seed samples. However, given the next question you raised, we have deleted this sentence to make this whole paragraph clearer. The result of the deletion is as follows:

~~‘and a portion of the samples was used for seed germination determination, while the remaining samples were immediately frozen in liquid nitrogen and stored in a -80°C refrigerator for the determination of other indicators.’~~

**24. Comment:** L138-148: the order or the information should be tweaked for clarity.

**24. Reply:** Thank you very much for your comments. Based on your comments, we have made some deletions and ordering adjustments to these sentences (L133-148).

**The deleted sentences are as follows:**

~~‘and a portion of the samples was used for seed germination determination, while the remaining samples were immediately frozen in liquid nitrogen and stored in a -80°C refrigerator for the determination of other indicators.’~~

**The order of the sentences is adjusted as follows:**

According to the following formula (1), the moisture content of the seed lot during desiccation was calculated by the weighing method [39].

Calculation formula of moisture content (MC) [39]:

$R=\frac{M2-M1\times\left( 1-A \right)}{M2}\times100\%$(1)

where: R is the seed moisture content, %; M1 is the mass of the undried seed, g; M2 is the mass of the dried seed, g; A is the initial moisture content of the undried seed, %.

Samples of the seeds were randomly taken under different drying treatments (37.06%, 33.99%, 30.63%, 28.11%, 25.09%, 22.04%, 19.08%, 16.04%, 13.05%, and 9.66%). Fresh undried (37.06%) seeds were used as the control group. A thermo-hygrometer was placed on the shelf to record the daily temperature and humidity in the room throughout the experiment. During the desiccation process, all experimental seeds were regularly mixed to ensure uniform drying. During each stage of drying (including fresh seeds), four replicates of 100 seeds each were taken for the determination of seed germination, and three replicates of 40 seeds each were taken for the determination of the remaining indicators. The seed drying rate index (k) was calculated by dividing the amount of water loss on a wet basis (%) by the corresponding elapsed time in hours [40].

**25. Comment:** L 155- did any studies show these seeds have dormancy, or is it your assumption? If this is your unpublished result, you should probably include this in the introduction.

**25. Reply:** Thank you very much for your comments. **The studies of Xiaohua Shi and Zhongli Shi reported the existence of dormancy in the seeds of *P. chekiangensis* [7, 8]**, and we have also done pre-experiments on the subject, and our results also concluded that dormancy exists in the seeds of *P. chekiangensis*. We apologize for not including the information in the manuscript, **and after careful consideration, we have decided to add it to the introduction so that readers can have a clearer understanding. The results of the revision are as follows:**

**‘**In addition, there is dormancy in the seeds of *P. chekiangensis*, and variable temperature stratification can break the dormancy better [7, 8].**’**

**References:**

7.	Shi X-H, Shi Z-L. Preliminary study on dormancy physiology of *Phoebe chekiangensis* seeds. Journal of Zhejiang Forestry College. 1990; 7(4):85-90.

8.	Shi X-H, Shi Z-L. Study on Seed and Dormancy of *Phoebe chekiangensis* and Its Releasing Ways. Seed. 1990; (3):76. <https://doi.org/10.16590/j.cnki.1001-4705.1990.03.073>

**26. Comment:** L 156-160- a lot going on. Did you use 15/25C for the dormancy break? Why did you choose this temperature range? It is also unclear why 24-hour light was chosen for germination.

**26. Reply:** Thank you very much for your comments. This question is related to the previous one. We referred to the research results of Xiaohua Shi and Zhongli Shi and found that 15 / 25°C can better break the seed dormancy of *P. chekiangensis*. We chose two temperature ranges (15 °C 16h / 25°C 8h and 15°C 16h / 30°C 8h) to do the pre-experiment, and we also found that the temperature range of 15 / 25°C can break the seed dormancy better. We think your suggestion is very good and thank you again for your constructive comments.

During the pre-experiment we did, the germination of the seeds was set up with 24 hours of light, and we found that the final germination results were good, and for this experiment to ensure the consistency of the experimental conditions, we still chose 24 hours of light to germinate. However, during the experiment, we felt that the germination conditions of *P. chekiangensis* seeds are not harsh, and the seeds may not need 24 hours of light conditions to germinate well. We think your question is very good, and we will continue to explore the germination conditions of *P. chekiangensis* seeds, especially the light conditions, in our future research. Thanks again for your comments.

**27. Comment:** For sections 2.3 and 2.4, you mentioned the methodology but did not give any details about the materials. It is seeds, but what treatments were done to them is not given. This information is crucial.

**27. Reply:** Thank you very much for your comments. Based on your comments, we added some information about the experimental material in the revised manuscript of L278, as follows:

‘Sample pre-treatment: we took the seed samples from the -80 °C refrigerator, removed the seed coat, and then put every 40 seeds (one repeat) into a low-temperature grinder to grind into powder, and mixed the powder of 40 seeds into a 50ml centrifuge tube and then placed it in liquid nitrogen for temporary preservation. After all the samples were treated in this way, the 27 (9 treatments, 3 replicates per treatment) centrifuge tubes filled with powder were placed in a refrigerator at -80 °C for testing.’

When weighing the sample, we use the weighing spoon to quickly mix the sample in the centrifuge tube and then weigh the required grams to carry out the next experiment. For section 2.4, we would add a sentence (L308 in the revised manuscript) as follows:

‘Extraction of the sample: Sample pre-treatment as above.’

**28. Comment:** L 217- Control seeds?

**28. Reply:** Thank you very much for your comment. Our presentation is not clear here. The samples we measured were not just the control group, but all seed samples. Based on your comments, and for the better understanding of the reader, we have made the following modifications to the sentence (L217):

**‘All seed samples were used as experimental determinations.’**

**29. Comment:** L 233- what is MDA?

**29. Reply:** Thank you very much for your comment. MDA in the manuscript refers to malondialdehyde. We apologize that we did not express this matter clearly, and we added a clarification to this in the revised manuscript, which is presented below:

**The malondialdehyde (MDA) content** was determined with some modifications according to the method of Chen et al. [45].

**30. Comment:** L 246- so you did both square root and log transformation? Whilst which transformation is better could be a subject of unresolved debate, all researchers would agree that a consistent transformation should be followed.

**30. Reply:** Thank you very much for your comments. We agree that the data should follow a consistent conversion. Therefore, based on your comments, we performed a natural logarithm conversion of germination percentage data. **Among them, the data modification will involve the change of the result part and supplementary material. Please see the revised manuscript (L409-439 and L581-624 in the revised manuscript) and supplementary material (S1 Fig) for more details.**

**In addition, we have deleted the sentence (L246) as follows:**

**~~The seed germination percentage was subjected to inverse sine square root transformation to meet the normal distribution and homogeneity of variance.~~**

**31. Comment:** L 272- after drying, the viability loss occurred. However, Jaganathan et al. (2019) said the seeds can become dormant during drying. Indeed, you mentioned something about dormancy but did not test this.

**31. Reply:** Thank you very much for your comments. As I mentioned in the revised manuscript of L258, the seeds of *P. chekiangensis* have dormancy and we described how to break the dormancy. **Instead of breaking the dormancy and then doing the desiccation experiment, we did the desiccation experiment immediately after the seeds were harvested, and then all seed samples were stratified at variable temperatures to break the dormancy. After breaking seed dormancy, we did the germination experiment, which ensured that the seeds were germinated. In this way, we made it clear that the loss of seed viability was due to desiccation rather than dormancy.** We think this is a good question and thank you again for your constructive comments.

Also, regarding the dormancy of the seeds of *P. chekiangensis*, we referred to several papers and did a pre-experiment. The seed dormancy of *P. chekiangensis* could be released by variable temperature stratification (15℃ 16 h / 25℃ 8 h, without light) for 60 days [7, 8].

**References:**

7. Shi X-H, Shi Z-L. Preliminary study on dormancy physiology of *Phoebe chekiangensis* seeds. Journal of Zhejiang Forestry College. 1990; 7(4):85-90.

8.	Shi X-H, Shi Z-L. Study on Seed and Dormancy of *Phoebe chekiangensis* and Its Releasing Ways. Seed. 1990; (3):76. <https://doi.org/10.16590/j.cnki.1001-4705.1990.03.073>

**32. Comment:** L 281- what is semi-lethal?

**32. Reply:** Thank you very much for your comment. ‘semi-lethal’ means that the germination percentage of the seed has dropped to 50%. Our expression is indeed not very suitable, so we have made the following modifications:

**‘According to this equation, the moisture content of *P. chekiangensis* seeds is 29.05% when the germination percentage drops to 50%.’**

**33. Comment:** L 295- Perhaps a better approach could include such analysis during seed development, at least from the maturation drying stage, i.e., when the pericarp colour starts to change.

**33. Reply:** Thank you very much for your comments. We agree that it would be better to do such an analysis to include the period of seed development. However, currently, the research of our teams focuses on the response of seeds to desiccation after maturation, including physiological and biochemical aspects. We think this is a very constructive comment, and we will include the period of seed development in our future studies, to better explore the mechanism of recalcitrance in *P. chekiangensis* seeds. Thank you again for your excellent comments.

**34. Comment:** L 416- For *P. chekiangensis* seeds. What is this- this statement is incomplete.

**34. Reply:** Thank you very much for your comment. We apologize that sentence L146 does have a problem with the presentation, and based on your comment, we have made the following modifications:

**‘The moisture content of the seeds of *P. chekiangensis* was also higher (37.06%) when they were matured and shed,’**

**35. Comment:** L420- classifying them as recalcitrant seeds- there is something wrong with the whole sentence and expression.

**35. Reply:** Thank you very much for your comments. We apologize that the expression of the whole sentence is indeed problematic, and based on your comments, we have made the following modifications to the whole sentence:

‘The moisture content of the seeds of *P. chekiangensis* was also higher (37.06%) when they were matured and shed, and the seeds are sensitive to desiccation, seed germination percentage decreased to 5.33% when the moisture content decreased to 22.04% (Fig 1C), therefore, the seeds of *P. chekiangensis* are recalcitrant.’

**36. Comment:** L 434- I also think such variation might be due to family-level variation. Therefore, if possible, can you compare the seed coat structure and size of all these seeds in relation to the species studied here?

**36. Reply:** Thank you very much for your comment, we think it is a constructive one. However, this manuscript focuses on the changes in germination percentage and sugar compounds of seeds during desiccation, and there is no information on seed size and seed coat structure of *P. chekiangensis* in the results. It would be a bit abrupt if we discussed this information. Therefore, considering the overall logic of the manuscript, we deleted the sentence L432-433 (see purple font below ①). We do intend to collect information about the seed size and the seed coat structure, and we hope to publish the results of our study in the future. We hope that our response will satisfy you and thank you again for your excellent comment.

① ~~‘These differences in sensitivity to desiccation may be related to variations in seed size, seed coat structure, and inclusions of the seed embryo.’~~

**37. Comment:** L442-462- The discussion of the resurrection plant is not necessary, in my opinion. You merely have to state this is the case in vegetative tissues and focus your attention on seeds.

**37. Reply:** Thank you very much for your comments. We agree that the discussion of the resurrection plant here is unnecessary, and therefore we deleted this section (L442-462) and focused the discussion primarily on seeds. We revised the discussion section (L694-L761) to read as follows:

Previous studies have shown a correlation between sugar accumulation and seed desiccation tolerance [49-52]. Mechanisms to protect against desiccation damage may exist in both the vegetative organs and seeds of desiccation-tolerant plants, such as the accumulation of sucrose and raffinose family oligosaccharides (raffinose and stachyose), which help stabilize membranes, proteins, and the cytoplasmic glass matrix [53]. **How do the sugars of recalcitrant seeds respond to desiccation? During desiccation, the contents of sucrose, trehalose, and soluble sugar in *P. chekiangensis* seeds increased significantly, and the starch content decreased. This is consistent with the findings of desiccation-sensitive *Inga vera* seeds [54]. Hydrolysis of starch may contribute to the increase in sugars. Moreover, the increase in soluble sugars observed in the seeds of *P. chekiangensis* may also be a result of reduced seed respiration rate, which reduces sugar consumption. Correlation analysis revealed that the accumulation of sucrose and trehalose was associated with reduced seed viability, indicating that the accumulation of the sugars acts as a protective substance in the seed, but low levels of sugar accumulation still do not prevent the loss of seed viability.** Notably, it has also been argued that the impact of sugar on seed desiccation tolerance is not isolated but rather connected to both the developmental stage of the seed and the synergistic effects of abscisic acid and proteins [18].

**38. Comment:** L 475- From what is presented here, I don’t see any methods for evaluating the raffinose or other sugar during maturation.

**38. Reply:** Thank you very much for your comments. The main purpose of our manuscript is to explore the changes in sugar compounds during desiccation. At present, it does not involve sugar metabolism during maturation. Therefore, considering the integrity of the manuscript, we did not express this content in the discussion. We hope that we can do more research on the seed maturation process in the future. In addition, we've removed and rewritten this part of L442-482. We hope you are satisfied with our reply and thank you again for such excellent comments.

**39. Comment:** The authors also speculate too much in the discussion and often mix desiccation-tolerant species results with the present study. It would be good if the authors toned down their arguments and placed more in the context of their results.

**39. Reply:** Thank you very much for your comments. In response to your comments, we have removed as much as possible some of the overly diffuse hypothetical conclusions from the discussion and kept a section of reasonable hypotheses. We agree that it is not quite appropriate to often mix the results of desiccation-tolerant species with the present study, so we removed this section from the discussion. Finally, based on your comments, we revised the discussion section (L692-775) to focus more on our results. Thank you again for such excellent comments. The revised discussion section is read as follows:

**Response of sugars as the protective substance to seed desiccation**

Previous studies have shown a correlation between sugar accumulation and seed desiccation tolerance [49-52]. Mechanisms to protect against desiccation damage may exist in both the vegetative organs and seeds of desiccation-tolerant plants, such as the accumulation of sucrose and raffinose family oligosaccharides (raffinose and stachyose), which help stabilize membranes, proteins, and the cytoplasmic glass matrix [53]. **How do the sugars of recalcitrant seeds respond to desiccation? During desiccation, the contents of sucrose, trehalose, and soluble sugar in *P. chekiangensis* seeds increased significantly, and the starch content decreased. This is consistent with the findings of desiccation-sensitive *Inga vera* seeds [54]. Hydrolysis of starch may contribute to the increase in sugars. Moreover, the increase in soluble sugars observed in the seeds of *P. chekiangensis* may also be a result of reduced seed respiration rate, which reduces sugar consumption. Correlation analysis revealed that the accumulation of sucrose and trehalose was associated with reduced seed viability, indicating that the accumulation of the sugars acts as a protective substance in the seed, but low levels of sugar accumulation still do not prevent the loss of seed viability.** Notably, it has also been argued that the impact of sugar on seed desiccation tolerance is not isolated but rather connected to both the developmental stage of the seed and the synergistic effects of abscisic acid and proteins [18].

The supply of hexoses (glucose and fructose) during desiccation is an important step in the regulation of sugar biosynthesis [55]. In our desiccation experiments, we observed that fructose and glucose predominated among soluble carbohydrates, and they showed a slight increase in the middle of desiccation but significantly decreased towards the late desiccation stage, which is in general agreement with the results of Ghasempour et al. [56] and Whittaker et al. [55]. We speculate that the slight increase in glucose and fructose in the middle of desiccation may be attributed to a higher rate of starch decomposition compared to sucrose synthesis at this stage. In addition, the transient accumulation of these sugars in the middle of desiccation contributes to osmoregulation [56]. We also found that the raffinose content of *P. chekiangensis* seeds increased during the later stages of desiccation (S1 Table), in contrast to the results for desiccation-sensitive *Inga vera* seeds [54]. The accumulation of high levels of hexoses and low levels of sucrose and oligosaccharides may be a characteristic of some desiccation-sensitive seeds.

**40. Comment:** Figure orders should be adjusted in the manuscript.

**40. Reply:** Thank you very much for your comment. Based on your comment, we have decided to move Fig 6 from the original manuscript to the Supporting Information, while the original Fig 7 has become Fig 6.

**Reviewer 2:**

**1. Comment:** The authors provided a precise study of the natural drying of seeds of endangered Chinese species *Phoebe chekiangensis*. Seeds seemed to be recalcitrant as most of them usually do not survive moisture content lower than 29%. The analysis of carbohydrate profile, content of starch and MDA, relative electrical conductivity and germination tests with precise statistical evaluation of obtained data would bring an interesting conclusion that the accumulation of reducing sugars fructose and glucose instead of non-reducing sugars sucrose and RFO (stachyose were not detected at all). Unfortunately, these data are not fully reliable, if you measured dead seeds (as you mentioned most of the seeds were dead at the moisture content below 28%) and if you calculate all values to fresh weight instead of dry weight. This is very important if the main changing parameter is moisture content.

**1. Reply:** Thank you very much for your comments, which we think are very constructive. As you said, we found that more than half of the seeds died when the moisture content was below 28%, however, we believe that the study of seeds is a population study and that even if the germination percentage is below 50%, it does not mean that all the seeds are dead and there are still viable seeds. We sampled three replicates of each treatment, each replicate with 40 randomly selected seeds, and we ground and mixed all 40 seeds in each replicate, and then weighed the samples of the grounded seeds for determination. It is really easy to get dead seeds if only 3 or 5 seeds are taken in each replicate, and we sampled a larger number of seeds in each replicate to avoid the above situation as much as possible. Furthermore, theoretically, all the seeds are dead when the germination percentage is 0. Our understanding of 0 germination percentage is that the seeds cannot germinate, but the internal physiological activities of such seeds may still exist for a short time, so we believe that the measurement of the physiological and biochemical indexes when the germination percentage is 0 is also of some reference significance. In addition, we also found a similar situation in the study of Karin et al. [46].

Concerning the comment on the fresh and dry weight, we believe that it is a constructive one. After careful discussion, we considered converting the parameters like sugar compounds in the original manuscript to dry weight-based units, such as mg/g FW to mg/g DW. When calculating this, the moisture content of each desiccation treatment is known, and theoretically, we can calculate the dry weight of the samples for each treatment based on the moisture content and the fresh weight of the samples. Theoretically, we believe that this conversion can eliminate the difference in moisture content between the different drying treatments. After such conversion, we found that the trends of the data before and after conversion were consistent in general, as shown in Figs. (A-D, A1-D1), so we thought that the determination of the parameters based on fresh weight was also informative, and we chose to keep the data from the original manuscript. We think this is a very good opinion, and we will consider your comments to make our research even better in the future. Finally, we hope you are satisfied with our response, and thank you again for your comments.

Note: Figs. A-D is the data before modification and Figs. A1-D1 is the data after modification. Fresh weight, FW. Dry weight, DW.

**References:**

46.	van der Walt K, Burritt DJ, Nadarajan J. Impacts of Rapid Desiccation on Oxida- tive Status, Ultrastructure and Physiological Functions of *Syzygium maire* (Myrtaceae) Zygotic Embryos in Preparation for Cryopreservation. Plants. 2022; 11(8):1056. <https://doi.org/10.3390/plants11081056> PMID: [35448783](https://pubmed.ncbi.nlm.nih.gov/35448783)

**2. Comment:** In the description of methods, I would recommend to join into one chapter these chapters: 2.4, 2.4.1., 2.4.2. and 2.4.3. The last one is written like a protocol in the student notebook, please try to restyle into phrases. Chapter 2.5. could be divided into two chapters and not two subchapters.

**2. Reply:** Thank you very much for your comments. Based on your comments, we have combined the following chapters into one: 2.4, 2.4.1, 2.4.2, and 2.4.3 (L302, 308, 314, and 323). Regarding the last one, we have also reorganized it into one paragraph based on your comments (see the purple bolded font below ①). In addition, chapter 2.5 has been split into two chapters based on your comments.

**① ‘GCMS operating conditions: the instrument was a gas chromatography triple quadrupole tandem mass spectrometer, model TSQ 9000 AEI (GCMSMS). HP-5 chromatographic column (30 m × 0.25 mm × 0.25 μm), injection volume was 1 μL, gasification chamber temperature was 280°C, split ratio was 100:1, carrier gas was helium (99.999%), and flow rate was 1 mL·min^-1^. The initial temperature of the chromatographic column was 80°C for 1 min, and the temperature was increased to 240°C at a rate of 6°C·min^-1^ for 15 min.’**

**3. Comment:** Last but not least, I would recommend to submit this manuscript to some journal more releavnt to the topic and used methods, e.g., Seed Science and Technology.

**3. Reply:** Thank you very much for your comment, we think it is a constructive one. The research direction of our manuscript is about seeds, and it would be good to submit it to a journal with a more relevant topic such as Seed Science and Technology, but Plos One is also trustworthy as a comprehensive journal, and because of the trust in this journal and the consideration of the submission cycle, we are willing to submit it to this journal in the end. We hope to get your understanding and we will consider more relevant journals based on your comments if we have more manuscripts on this topic in the future.

**Reviewer 3:**

**1. Comment:** The manuscript is well written but still have some grammatical errors such as: L 352. during this period, the seeds experienced severely damaged due to drying, with or L 416. The manuscript needs to be revised for such errors.

**1. Reply:** Thank you very much for your comments, we apologize for these grammatical issues. Based on your comments, we have modified the sentence L352 as follows:

**‘Notably, both RC and MDA contents increased significantly (*p* < 0.05) when the seeds were dried to 28.11% MC, indicating that at this stage the seeds began to suffer damage.’**

Based on your comments, we have also modified the L416 sentence as follows:

**‘The moisture content of the seeds of *P. chekiangensis* was also higher (37.06%) when they were matured and shed,’**

**2. Comment:** L 133. The formula should be provided at the end of this sentence.

**2. Reply:** Thank you very much for your comment, based on your comment, we have moved the formula directly after the sentence L133 and numbered the formula. The result of the modification is as follows:

**According to the following formula (1), the moisture content of the seed lot during desiccation was calculated by the weighing method [39].**

**Calculation formula of moisture content (MC) [39]:**

$R=\frac{M2-M1\times\left( 1-A \right)}{M2}\times100\%$(1)

**where: R is the seed moisture content, %; M1 is the mass of the undried seed, g; M2 is the mass of the dried seed, g; A is the initial moisture content of the undried seed, %.**

**3. Comment:** L 311. % trehalose content increase (23.03) under drying seemed to be wrong. See Fig 2C

**3. Reply:** Thank you very much for your comment. We apologize for the mistake, based on your comments, we have made the following modification to sentence L311:

‘Ultimately, **the trehalose content increased about 23-fold** when the seeds dried to 13.05% MC.’

**4. Comment:** L 332-338. Concluding remarks (based on Fig 3) should be more precise and should be based on data obtained. Modify these sentences to draw more meaningful conclusions. Also check Fig 3C. It needs more clarity especially w.r.t. RS/NRS. Is it ratio of RS to NRS or something else?

**4. Reply:** Thank you very much for your comments. Based on your comments, we have added corresponding data to our results and tried to produce more meaningful results wherever possible. We have therefore modified the paragraph L332-338 as follows:

We found that the specific non-reducing sugar content (the sum of sucrose, trehalose, and inositol content) increased during drying, with an approximately 82-fold increase in the non-reducing sugar content at 13.05% MC (Fig 3A). In contrast, **the ratio of (glucose + fructose + arabinose + galactose) to (sucrose + trehalose + inositol) (RS/NRS)** was decreased during drying, with the ratio decreasing by almost 1-fold at 13.05% MC (Fig 3C). Among the three non-reducing sugars (sucrose, trehalose, and inositol), sucrose was the most abundant, while glucose and fructose dominated among the four reducing sugars (Fig 3A and 3B).

Based on your comments, we examined Fig 3 and did find some problems, and we have made modifications as follows:

Fig 3. Effects of different moisture contents on (A) non-reducing sugar (NRS), (B) reducing sugar (RS), and (C) reducing sugar/non-reducing sugar (RS/NRS) during seed desiccation. The non-reducing sugar content refers to the combined amount of sucrose, trehalose, and inositol, whereas the reducing sugar content refers to the combined amount of fructose, glucose, galactose, and arabinose. **RS/NRS refers to the ratio of (glucose + fructose + arabinose + galactose) to (sucrose + trehalose + inositol).** One-way ANOVA analysis was performed on the data. Different lowercase letters represent significant differences at the *p* < 0.05 level according to the Duncan test. Data represent mean ± SE.

In addition, RS/NRS in Fig 3 refers to the ratio of (glucose + fructose + arabinose + galactose) to (sucrose + trehalose + inositol). I'm sorry we didn't make it clearer, and we have labeled them in the revised manuscript, as detailed in the purple bolded font above. Thanks again for your comments.

**5. Comment:** L 389 – L 395. The relevance of correlation of germination with different parameters can be made more meaningful if glucose, fructose, galactose etc are also compared with germination. The Fig 6 failed to present meaningful information as it did not include data on above mentioned parameters. Moreover, there is no mention of positive and negative correlation. The Fig 5 and Fig 6 are almost similar and hence, one of the figures may be included as supplementary figure.

Similarly, for Fig 7, only those parameters that are significantly correlated to germination should be included for more meaningful presentation.

**5. Reply:** Thank you very much for your comments. We have made changes based on your comments and the changed figure included the relationship between germination percentage and soluble sugars, trehalose, sucrose, galactose, and fructose. The relationship between germination percentage and glucose was not significant, so this result was not put into the figure. In addition, the positive and negative correlations between the parameters have been expressed in the correlation analysis (Fig 5), thus the linear regression analysis did not focus on this.

Based on your suggestion, we have placed Fig 6 from the original manuscript as a supplemental figure (S1 Fig) in the Supporting information.

**S1 Fig. Linear regression analysis between germination percentage and some sugar compounds.** The regression line is represented by the black line. GP: germination percentage, SS: soluble sugar.

For Fig 7 in the original manuscript, we strongly agree with your proposal, however, the parameters we chose were the ones that were significantly correlated with germination percentage, and based on that, we ended up keeping them. Thank you again for your constructive comments.

**6. Comment:** Discussion and conclusion parts also need refinements especially with respect to resurrection plants and recalcitrant plants. If sugar patterns are similar in both under drying, the present study apparently failed in its objective of understanding mechanisms leading to reduced germination under drying in the recalcitrant plant.

**6. Reply:** Thank you very much for your comments. Based on your comments, we have modified the discussion and conclusion section by deleting the related content of the resurrection plants and concentrating on the seeds and our results. We present the revised discussion and conclusions below:

**Response of sugars as the protective substance to seed desiccation**

Previous studies have shown a correlation between sugar accumulation and seed desiccation tolerance [49-52]. Mechanisms to protect against desiccation damage may exist in both the vegetative organs and seeds of desiccation-tolerant plants, such as the accumulation of sucrose and raffinose family oligosaccharides (raffinose and stachyose), which help stabilize membranes, proteins, and the cytoplasmic glass matrix [53]. **How do the sugars of recalcitrant seeds respond to desiccation? During desiccation, the contents of sucrose, trehalose, and soluble sugar in *P. chekiangensis* seeds increased significantly, and the starch content decreased. This is consistent with the findings of desiccation-sensitive *Inga vera* seeds [54]. Hydrolysis of starch may contribute to the increase in sugars. Moreover, the increase in soluble sugars observed in the seeds of *P. chekiangensis* may also be a result of reduced seed respiration rate, which reduces sugar consumption. Correlation analysis revealed that the accumulation of sucrose and trehalose was associated with reduced seed viability, indicating that the accumulation of the sugars acts as a protective substance in the seed, but low levels of sugar accumulation still do not prevent the loss of seed viability.** Notably, it has also been argued that the impact of sugar on seed desiccation tolerance is not isolated but rather connected to both the developmental stage of the seed and the synergistic effects of abscisic acid and proteins [18].

The supply of hexoses (glucose and fructose) during desiccation is an important step in the regulation of sugar biosynthesis [55]. In our desiccation experiments, we observed that fructose and glucose predominated among soluble carbohydrates, and they showed a slight increase in the middle of desiccation but significantly decreased towards the late desiccation stage, which is in general agreement with the results of Ghasempour et al. [56] and Whittaker et al. [55]. We speculate that the slight increase in glucose and fructose in the middle of desiccation may be attributed to a higher rate of starch decomposition compared to sucrose synthesis at this stage. In addition, the transient accumulation of these sugars in the middle of desiccation contributes to osmoregulation [56]. We also found that the raffinose content of *P. chekiangensis* seeds increased during the later stages of desiccation (S1 Table), in contrast to the results for desiccation-sensitive *Inga vera* seeds [54]. The accumulation of high levels of hexoses and low levels of sucrose and oligosaccharides may be a characteristic of some desiccation-sensitive seeds.

**Conclusions**

In this study, we analyzed the effects of different drying treatments on seed germination, sugar compounds, MDA, and relative electrical conductivity in *P. chekiangensis* seeds. The initial moisture content of the seeds was high (37.06%) and the seeds were sensitive to desiccation, for example, when the seeds were dried to 22.04% MC, they lost 94% of their viability, therefore, the seeds of *P. chekiangensis* were considered to be recalcitrant. According to the logistic model, we know that when seed germination drops to 50%, the moisture content is 29.05%, and the MC should ideally be kept above 31.74% when the seed lot is transported at room temperature. The contents of sucrose and trehalose tended to increase during seed desiccation. Correlation analyses showed that the reduction in seed viability was significantly correlated with the accumulation of sucrose, trehalose, and soluble sugars, suggesting that the sugar acts as a protective substance against desiccation, but low levels of the sugar accumulation were still unable to inhibit the loss of seed viability induced by desiccation. We observed that among the sugar compounds, glucose and fructose were predominant, with a slight increase in their content in mid-desiccation and a significant decrease in late desiccation. The accumulation of glucose and fructose in the middle stage of desiccation is beneficial for osmoregulation. Both MDA and relative electrical conductivity showed an increasing trend during seed desiccation, indicating potential damage to the membrane system induced by seed drying.

**7. Comment:** To draw meaningful conclusions, the seeds of *P. chekiangensis* must be rehydrated following drying to see if those resume physiological activities including germination following desiccation or not. Appropriate tables and figures related to this experiment may then be added to the manuscript.

**7. Reply:** Thank you very much for your opinion, we think this is a very constructive one. The seeds of *P. chekiangensis* were dehydrated and then rehydrated to observe whether the seeds had restorative physiological activities after rehydration. This opinion can help us to study the recalcitrance of *P. chekiangensis* seeds more deeply. However, our current experiment is mainly focused on the physiological response of the seeds to desiccation. We believe that this experiment is also meaningful, and Feng et al. have also studied the physiological response of *Ginkgo biloba* seed during desiccation [39]. However, it is a pity that the research on rehydration after dehydration has not been carried out at present. We hope that this research can be carried out in the future so that we can have a deeper understanding of the recalcitrance of *P. chekiangensis*. We hope our answers will satisfy you and thank you again for your excellent comments.

**References:**

39.	Feng J, Shen Y, Shi F, Li C. Changes in seed germination ability, lipid peroxidation and antioxidant enzyme activities of *Ginkgo biloba* seed during desiccation. Forests. 2017; 8(8):286. <https://doi.org/10.3390/f8080286>
